# Skeletal Muscle in Hypoxia and Inflammation: Insights on the COVID-19 Pandemic

**DOI:** 10.3389/fnut.2022.865402

**Published:** 2022-04-22

**Authors:** Filippo G. Di Girolamo, Nicola Fiotti, Ugo G. Sisto, Alessio Nunnari, Stefano Colla, Filippo Mearelli, Pierandrea Vinci, Paolo Schincariol, Gianni Biolo

**Affiliations:** ^1^Department of Medical Surgical ad Health Science, Clinica Medica, Cattinara Hospital, University of Trieste, Trieste, Italy; ^2^SC Assistenza Farmaceutica, Cattinara Hospital, Azienda Sanitaria Universitaria Giuliano Isontina, Trieste, Italy

**Keywords:** hypoxia, sarcopenia, sarcopenic obesity, inflammation, oxidative stress

## Abstract

SARS-CoV-2 infection is often associated with severe inflammation, oxidative stress, hypoxia and impaired physical activity. These factors all together contribute to muscle wasting and fatigue. In addition, there is evidence of a direct SARS-CoV-2 viral infiltration into skeletal muscle. Aging is often characterized by sarcopenia or sarcopenic obesity These conditions are risk factors for severe acute COVID-19 and long-COVID-19 syndrome. From these observations we may predict a strong association between COVID-19 and decreased muscle mass and functions. While the relationship between physical inactivity, chronic inflammation, oxidative stress and muscle dysfunction is well-known, the effects on muscle mass of COVID-19-related hypoxemia are inadequately investigated. The aim of this review is to highlight metabolic, immunity-related and redox biomarkers potentially affected by reduced oxygen availability and/or muscle fatigue in order to shed light on the negative impact of COVID-19 on muscle mass and function. Possible countermeasures are also reviewed.

## Introduction

SARS-CoV-2 infection causes severe inflammation and oxidative stress leading to pulmonary alveoli damage. This in turn can lead to bilateral viral pneumonia with respiratory failure and development of severe acute respiratory distress syndrome (ARDS) (1–4). Several meta-analytic studies showed that myalgia/muscle fatigue are the most common symptoms (after fever and cough) of COVID-19 infection (5–9). Moreover, the extent of COVID-related myalgia seems to depend mainly on the severity of the disease (9, 10). In fact, radiological data from hospitalized COVID-19 patients suggest that the correlation between muscle pain on admission and abnormal lung imaging is a predictor of poor prognosis, particularly in the elderly patients (11). The exact mechanisms by which SARS-CoV-2 causes muscle damage are not yet fully elucidated (4). Some authors propose that muscle loss in COVID-19 patients is the result of a wide variety of factors, both direct (e.g., interaction between the virus spike proteins and myocyte cell membranes) and indirect (inflammation and oxidative stress) (4). Indeed, oxidative stress and inflammation are the two most relevant drivers of skeletal muscle loss (5, 6, 12, 13).

It is important to consider that the elderly population is one of the most heavily affected by SARS-CoV-2. Prevalence of protein-energy malnutrition and chronic systemic inflammation is increased with aging, metabolic disorders and non-communicable diseases (i.e., obesity, diabetes mellitus, cardiovascular diseases, cancer, etc.) (14, 15). These conditions are also risk factors for severe COVID-19 (16). While systemic muscle atrophy has been reported in COVID-19 patients during home treatment, greater “disease-related malnutrition” has been observed during hospitalization, particularly in intensive care units (ICUs). The most extensive muscle loss is found in obese patients (17, 18). A number of factors may promote muscle weakness in COVID-19 patients: (1) long period of bed rest (19–22); (2) some drugs and medications (10, 23, 24); (3) malnutrition/undernutrition (22, 23); (4) ICU-acquired weakness (23, 25, 26); (5) severe cytokine storm (10, 23); (6) intubation and mechanical ventilation (4); and (7) long-COVID (19–21, 26). While the link between physical inactivity, chronic inflammation and oxidative stress and muscle dysfunction is well-established (27), the effect on muscle mass of COVID-19-related hypoxemia has been insufficiently evaluated. Indeed, hypoxemic chronic cardiopulmonary conditions (e.g., Chronic obstructive pulmonary disease—COPD-, pulmonary fibrosis, Obstructive Sleep Apnea Syndrome—OSAS—or heart failure), induce a hypoxia-related decreased physical capacity (28, 29). Systemic hypoxemia determines muscle and bone deterioration (30–32) by triggering several signaling cascades including innate immune responses and/or changes in redox balance and lipid and glucose metabolism (33, 34). Despite the fact that the combination of hypoxia and reduced physical activity has been frequently observed in patients, the interaction between these factors has been scarcely investigated in the clinical settings. In fact, the specific effects of inactivity and hypoxia are difficult to separate from other disease related variables.

Experimental BR is one of the most appropriate methods to study the consequences of immobilization in a standardized and controlled environment (35). This approach has been recognized to be of potential clinical relevance for mimicking hospital-related physical inactivity since it allows differentiating the catabolic effects induced by a disease from those secondary to skeletal muscle disuse (35). The study of healthy volunteers in facilities that allow a controlled modification of the amount of oxygen in the air in both bed rest and/or ambulatory conditions can be considered an appropriate model to investigate the separate and the combined effect of hypoxia and muscle disuse (28, 36, 37).

This review tries to identify some metabolic, immunity-related and redox biomarkers, potentially affected by decreased oxygen blood levels and/or muscle disuse (28, 38–40), to shed light on the negative impact of SARS-CoV-2 infection on muscle mass and function. Moreover, possible countermeasure are described.

Papers for this narrative review were obtained from Medline (PubMed), and Cochrane Library Plus database. The mesh terms utilized have been hypoxia, bed rest, inflammation, oxidative stress and dyslipidemia being conditions associated with COVID-19, SARS-CoV-2, Long COVID and COVID sequelae. Titles and abstracts were used to identify selected articles. More attention was payed to the most recent publications.

### Muscle Protein Metabolism

The dynamic balance between protein synthesis and degradation processes is the key factor behind maintenance and accumulation of muscle mass. When protein breakdown is higher than protein synthesis, there is a reduction in both skeletal muscle mass and myofibers cross-sectional area leading to decreased muscle strength and function (35, 41). Chronic hypoxia negatively affects skeletal muscle protein metabolism (42–44). A reduced protein synthesis rate was found in malnourished patients with emphysema (45). Animal models have shown that both protein synthesis and degradation are enhanced under hypoxic conditions, however, the rate of protein degradation is higher than that of protein synthesis (30, 46). Indeed, Agrawal et al. established that a week of both acute or chronic hypobaric hypoxia enhances Akt, p-Akt, and p70S6K expression, promoting protein synthesis. Such anabolic stimulus however decays by day 14 (30, 47).

Protein synthesis is an anabolic process, requiring an amount of energy which is insufficient in hypoxic conditions (48). Tissue protein synthesis is one of the most important determinant of whole body energy expenditure, therefore, tissue and cell energy status has a pivotal role on protein turnover kinetics. Oxidative metabolism, providing chemical energy for adenine-triphosphate (ATP) production, occurs in mitochondria. ATP is then used for all cellular and tissue functions (49). Altered mitochondrial function and impairment of oxidative capacity is a common feature of several catabolic conditions (50). The angiotensin-2 converting enzyme receptor (ACE-2) contributes to the regulation of mitochondrial functions. ACE-2 deficiency can develop both from a lower ATP synthesis and the activation of NADPH oxidase 4. This enzyme is involved in the production of ROS. It protects the cells by destroying pathogens or triggering apoptosis in case of infection (51). SARS-COV-2, to penetrate human host cells binds to ACE-2 through its spike glycoprotein (52). Hence, the availability of the ACE-2 enzyme for its physiological functions could be impaired, contributing to the development of COVID-19 symptoms (50). Besides binding to ACE-2 receptors, SARS-CoV-2 spike proteins can bind to the host cell membrane through the transmembrane serine protease 2 (TMPRSS2) (53). Another target of TMPRSS2 is the estrogen-related alpha nuclear receptor, which regulates the transcription of genes related to mitochondrial functions and energy homeostasis (51, 53, 54). Furthermore, mitochondrial function and their efficiency in ATP production are important factors in the regulation of protein synthesis and protein turnover rates. Stable-labeled amino acid infusion and tissue biopsy with isotopic precursor-product models represent a valuable method to investigate mitochondrial protein fractional synthesis *in vivo*. (49). Rooyackers et al. found a reduced mitochondrial protein fractional synthesis rate in skeletal muscle from elderly humans (55). In a rat model, reduced mitochondrial DNA content in skeletal muscle showed to be a feature of age-related sarcopenia (56). Modifications of mitochondrial metabolism and gene expression might be key mechanisms of age-related muscle alterations. Oxygen-dependent mitochondria ATP production is also a critical factor. If on one hand mitochondria respiration is strictly linked to ATP synthesis, on the other it is regulated by uncoupling proteins. They dissipate the H^+^ gradient generating heat, thereby reducing mitochondrial efficiency (49). Aging increases the expression of uncoupling proteins in liver and skeletal muscle (57), thus potentially decreasing ATP production and availability. These changes could account for the reduced protein synthesis and turnover (49).

A retrospective study (48) evaluating the interindividual response variability of the skeletal muscle oxidative function during normoxic (N-BR) and hypoxic (H-BR) bed rest, lasting 10 and 21 days, respectively, showed that mitochondrial respiration was reduced by both N-BR (25.0 and 15.7% after 10 and 21 days) and H-BR (13.0 and 19.8% after 10 and 21 days) with worsening of BR effect under hypoxic conditions after 10 days. These data suggest that hypoxia negative effect occurs in a retrograde way, as demonstrated by Agrawal et al. (30, 47).

Cell oxygen supply is determined by the oxyhemia, which in turn is linked to the hemoglobin concentration and the partial pressure of arterial oxygen. The latter can be reduced in chronic conditions characterized by impaired cardiopulmonary capacity, eventually leading to a diminished alveolar respiration. A lower hemoglobin concentration (i.e., anemia) may occur in several chronic catabolic disorders (49) and, in COVID-19 frail patients. This condition could indicate a reduced ability of hemoglobin to support the increased oxygen demand of peripheral tissues from the infection-related hypermetabolic state. Taneri et al., showed that compared to more treatable cases, severe COVID-19 patients had lower hemoglobin (−4.08 g/L, 95% CI−5.12; −3.05) and red blood cell counts (−0.16 × 10^12^/L, 95% CI−0.31; −0.014), while ferritin (−473.25 ng/mL, 95% CI 382.52; 563.98) and red cell distribution width (1.82%, 95% CI 0.10; 3.55) were higher (58).

The retrospective study by Salvadego et al. evaluated the effects of experimental physical inactivity alone or combined with hypoxia. Both conditions determined similar O_2_ uptake peaks (4.1 and 3.3% after 10 days; 4.5 and 8.1% after 21 days, respectively) and muscle fractional O_2_ extraction (5.9 and 7.3% after 10 days; 6.5 and 7.3% after 21 days). These data confirm again the effect of hypoxia after 10 days.

Minimizing the loss of skeletal muscle mass through an appropriate supply of energy and protein is the therapeutic goal of clinical nutrition against acute and chronic inflammatory diseases. Nevertheless, the minimum protein requirement, adequate to blunt, as much as possible, protein catabolism and the related anabolic resistance, in different acute and chronic inflammatory diseases, has been little studied (59). The suggested dietary protein/amino acid intake to obtain the lowest rate of protein catabolism in critical illness is about 1.2–1.5 g/kg body weight/day (60–62). The ESPEN society indicates that a higher protein/amino acid intake (1.0–1.3 g/kg/day) could be required in non-obese and obese COVID-19 patients (63, 64).

### Muscle Inflammation

Anabolic resistance and the related systemic skeletal muscle loss are the result of multiple factors such as a decreased physical activity, often associated with an inadequate food intake, obesity, inflammation, hormonal dysregulation and presence of other comorbidities (65).

A higher plasma concentration of inflammatory biomarkers (interleukin-6, IL-6, tumor necrosis factor alpha, TNF-α, and C-reactive protein, CRP) characterizes age-associated inflammation (66). In turn, inflammation reduces net muscle protein synthesis (66) by enhancing, among others, the TNF-α pathway, with known inhibitory effects on myogenesis, Furthermore it upregulates nuclear factor-kappa beta (NF-κβ), a key transcription factor in skeletal muscle atrophy (67–70).

Three factors seem to have a major role in the pathogenesis of COVID-19: (1) over-inflammation, (2) depression/inhibition of the immune system, and (3) proinflammatory cytokine spread (71). These features combined are responsible for lung damage and eventually fatal respiratory complications (71). Indeed, SARS-CoV-2 infection can trigger the so called “cytokine storm” also known as “cytokine release syndrome,” that is an abrupt increase in the levels of pro-inflammatory cytokines (IL-6, IL-12, IL-17, IL-18, IL33, TNF-α) and CRP (70). Such cytokine storm probably down-regulates innate and adaptive immunity against SARS-CoV-2 infection (71) and causes tissue disruption and muscle protein synthesis reduction in a synergistic way

These alterations can be observed during bed rest (in hospital or at home) and in the post-acute phase, either after recovery or during the so-called “long COVID” (64–67). Investigations on hypoxia signaling pathways have highlighted the linkage with inflammation (72) at a molecular level, while clinical studies have confirmed the contribution of NF-kB or of Hypoxia Inducible Factor-1α (HIF-1α) in various conditions (72, 73).

In hypoxia-associated diseases, NF-κB results in the expression of pro-inflammatory cytokines, which further activate NF-κB via a positive feedback mechanism (74). This NF-κB upregulation vicious circle probably contributes to the elevated pro-inflammatory cytokine expression even in COVID-19 patients (75). Even if the role of viral proteins in triggering this response is demonstrated, the molecular mechanisms for this activation are still unclear. Chia-Ming Su et al. identified ORF7a as the SARS-CoV-2 spike proteins activating NF-κB (74). Hypoxia should be considered as another strong trigger of inflammation. Cellular adaptations to hypoxia are based on HIF-1α factor transcription. This dimeric protein is inactive under normoxic conditions but is activated under hypoxia (72). In mammals, HIF-1α plays a critical role in the cell responses to systemic oxygen levels, by affecting metabolic pathways and inflammation (76, 77). Dysregulation of the HIF-1α pathway is associated with several pathologies including cancer and cardiovascular diseases (76). Moreover, HIF-1α is also involved in the regulation of the aging process (78). Activation of HIF-1α is predictable during SARS-CoV-2 disease. The viral protein ORF3a, which contributes to SARS-CoV-2 infection and mitochondrial damage, appears to play an important role in the regulation of HIF-1α by increasing its synthesis HIF-1α in turn, as suggested by the cellular model, can promote infection and inflammatory responses caused by SARS-CoV-2 (76).

CRP is a recognized biomarker of systemic inflammation and severe infection. COVID-19 systemic inflammation, as measured by CRP, correlates strongly with the disease severity and mortality (79). Moreover, a close association between CRP and hypoxemic respiratory failure has also been demonstrated (79).

A number of studies reported increased levels of Serum amyloid A (SAA) among patients with mild and severe COVID-19 (80) with a gradual SAA increase concomitant with the worsening of the clinical conditions (81). Higher serum SAA levels are found in critically ill patients and there is a positive correlation with the disease severity (80). In line with previous observations (72), the systemic inflammatory response (i.e., increased blood concentration of CRP and SAA) is observed in healthy young volunteers exposed to experimental hypoxia, either in ambulatory or bed rest conditions (28). Repeated measurements of both SAA and CRP levels during the disease time-course may aid in monitoring the extent and severity of pneumonia (80, 81). These data support the hypothesis that hypoxia *per se* could be a trigger of inflammation. However, being the role of systemic inflammation in the pathogenesis of SARS-CoV-2 infection not fully defined, the associations between inflammation (as measured by CRP or SAA) and prognosis could be defined, to a certain extent, as speculative (82). Presepsin is a new biomarker of innate immunity (83) possibly useful for early diagnosis of sepsis (84). Biolo et al. demonstrated that presepsin can be activated by hypoxia combined with bed rest, but not in the ambulatory conditions (28). This suggests a synergy between hypoxemia and immobilization in triggering inflammation. Presepsin has also been identified as a biomarker of SARS-CoV-2 infection severity (85). Presepsin levels become significantly elevated in SARS-CoV-2 infection and can be considered a prognostic factor for longer hospital stays. These data confirm the experimental results from Biolo et al. (28) and Fukada et al. (85).

IL-6 is a soluble protein with pleiotropic effects on immune response, systemic inflammation, and hematopoiesis. After its synthesis within the first phase of inflammation, IL-6 inducts a wide range of acute phase proteins production, including CRP, SAA, fibrinogen, and others (86). Conversely, IL-6 promotes the reduction of fibronectin, albumin, and transferrin synthesis (86). Inactivity *per se* leads to a low-grade systemic inflammation (66). An experimental bed rest lasting 14-days increased the levels of pro-inflammatory cytokines including IL-6 and CRP (27). Moreover, serum levels of IL-6, IL-6 receptor and CRP were found increased also in healthy volunteers who spent 3 days at an altitude higher than 3,400 m (72). These data suggest a role of hypoxia *per se* in activating the IL-6 cascade. It is generally accepted that during SARS-CoV-2 infection, high levels of IL-6 (>10 pg/mL) are the leading cause of the cytokine storm and multiple-organ failure seen in advanced cases (80). Consequently, IL-6 is becoming a sensitive prognostic biomarker during COVID-19 (74).

Tocilizumab is an anti-IL-6 receptor monoclonal antibody, approved for the treatment of several inflammatory diseases (87). A number of observational studies showed that tocilizumab could improve the outcome in COVID-19 patients with pneumonia (88–90) while other Authors reached more cautious conclusions. Indeed, the efficacy of this monoclonal antibody can vary according to disease severity and background standards of care (91–93). In particular, in hospitalized, not mechanically ventilated patients, tocilizumab reduces the need for ventilation support but it does not improve survival (94, 95).

Glucocorticoids can rapidly inhibit the release of inflammatory cytokines (e.g., IL-6, TNFα) (96). A randomized trial on COVID-19 therapy demonstrated that dexamethasone could improve survival, particularly in the most severely ill patients (24). However, iatrogenic hypercortisolaemia has been shown to increase the bed rest-related muscle mass loss when compared to bed rest alone (24). Therefore, dexamethasone may increase the risk of acute sarcopenia in fragile patients.

Glutamine is the most abundant aminoacid in the human body and it is maximally present in skeletal muscle. Beyond nutritional and plastic functions, the amino acid has also a key role in immunity and anabolic processes (13). It is categorized as a non-essential amino acid however since in catabolic conditions its synthesis rate is deficient compared to the requirements, it has been recognized as a conditionally -essential nutrient (96).

Parenteral glutamine supplementation has therefore been given in acute conditions to counteract the fall in muscle protein synthesis and improve nitrogen balance (96) and clinical outcome (97–99). Biolo et al. demonstrated that hypoxia during experimental bed rest, decreased plasma glutamine by about 4% (28). To date, no clinical trials have evaluated possible changes in glutamine plasma levels in COVID-19 patients. Nevertheless, a case-control study comparing 220 COVID-19 patients (51.2 ± 6.7 years) supplemented with glutamine with 230 unsupplemented COVID-19 patients (51.3 ± 8.2 years), comparable for gender, and clinical status, showed that L-Glutamine supplementation, in the early period of COVID-19 infection, boosted the immune system mainly through inhibition of inflammatory responses. This event lead to a shortened hospital stay and a lower need for ICU (100). Cengiz et al. obtained similar results (101).

### Muscle Oxidative Stress

Glutamine is also one of the three amino acids needed for glutathione (GSH) synthesis. GSH, a tripeptide formed by glutamate, glycine and cysteine, represents the most important body endogenous antioxidant system, being active mainly in the cell mitochondria and nucleus (102, 103). Intracellular glutamine as glutamate precursor is fundamental for the synthesis of GSH (99). Glutamine deficiency is therefore associated with decreased levels of GSH and higher reactive oxygen species (ROS) production (104).

Cells are exposed to oxidative stress in several clinical conditions including nutrient starvation and catabolic stress after trauma, surgery, sepsis, and infection (102). Glutathione is a critical factor in protecting cells against oxidative stress. Indeed, there is a relation, with reciprocal influences, between inflammation and increased oxidative stress.

The gamma-glutamyl cycle regulates both glutathione catabolism and re-synthesis. Total glutathione levels and GSH/GSSG ratio influence GSH redox capacity. Hypoxia conditions are able to stimulate both the production of reactive oxygen species and antioxidant responses, including intracellular glutathione (28). Experimental studies demonstrated that hypoxia when combined with bed rest directly decreased total whole blood glutathione concentration. Such decrease was paralleled by a reduction in the rate of glutathione synthesis supported by a lower glutamate-cysteine ligase activity (the key enzyme in GSH production) (28). Moreover, the GSH/GSSG ratio dramatically decreased suggesting that oxidative stress is higher than the antioxidants capacity.

Severe coronavirus infections were characterized by a GSH deficiency status. Guloyan et al. evaluated GSH plasma levels and ROS in four patients with laboratory confirmed SARS-CoV-2 infection (105). Patients with higher baseline levels of GSH showed a decrease in ROS and a shorter illness course while decreased GSH levels were associated with increased ROS and more severe symptoms (105). As cysteine is the limiting precursor of the GSH tripeptide synthesis, treatment with N-acetylcysteine (NAC) strongly stimulates GSH synthesis. Indeed, by improving the reduced-to-oxidized glutathione ratio (GSH/GSSG) NAC is the antidote to treat paracetamol intoxication (106). Moreover, NAC was found to lower inflammation in pneumonia (107). Nonetheless, even if NAC supplementation during SARS-CoV-2 infection shows some benefits there is low evidence of its efficacy (105, 106). To the best of our knowledge, only one randomized controlled trial was performed to evaluate the effect of NAC supplementation on patients with severe COVID-19 (108). Patients were randomly assigned either to NAC, at the dose of 21 g for 20 h, or to dextrose 5%. Requirement of mechanical ventilation was chosen as primary endpoint of the trial, while secondary goals included indices such as time of mechanical ventilation, ICU admission and length of stay and mortality. The trial showed no treatment effect on the evolution of severe COVID-19 infections (108). Another study followed two COVID-19 patients supplemented with NAC and GSH by the oral or intravenous route (109). Although the sample size was very small, researchers demonstrated an improvement in patients clinical conditions following GSH administration.

### Sarcopenic Obesity: Role of Muscle-Fat Crosstalk

Obesity in COVID-19 patients has been shown to be frequent and to worsen the disease prognosis (110). Unfortunately, especially in the ICU cases, only the BMI has been measured while body composition analysis has been rarely assessed (111). When such an evaluation has been made by computed tomography (CT), it showed mostly an increased visceral adipose tissue, considered an index capable of predicting the severity of the disease (112–115).

In non-COVID-19 ICU patients a reduced skeletal muscle mass measured by CT, resulted a risk factor for survival and need for mechanical ventilation. These data show that BMI cannot be considered an independent predictor of mortality compared to muscle mass (115). Furthermore, in non-COVID-19 ICU patients a low skeletal muscle area and radiodensity, considered indices of muscle quality, were associated with increased rates of infections (116).

Skeletal muscle and adipose tissue can communicate through a biochemical endocrine crosstalk through the release of inflammatory cytokines (i.e., myokines and adipokines, respectively). This crosstalk can determine the onset and/or exacerbation of inflammation and oxidative stress, with consequent hypercatabolism (117). This can lead to the so-called Sarcopenic obesity, in which loss of muscle mass, strength and function is associated with an increased fat mass (118) especially in aged and/or obese individuals. The pathogenesis of sarcopenic obesity is multifactorial (e.g., sedentary lifestyle, dietary disorders, insulin resistance, inflammation, oxidative stress, etc.) however since symptoms are non-specific, sarcopenic obesity goes often unsuspected and undiagnosed (119).

As already described, excessive cytokine levels directly damage, multiple organs and tissues, including skeletal muscle.

Sarcopenia is associated not only with loss of skeletal muscle function but also with alteration in carbohydrate and lipid metabolism especially when there is a concomitant condition of obesity (120). Overweight is often complicated by dyslipidemia, with increased serum levels of total cholesterol, fasting triglycerides (TG) and apolipoprotein B and decreased high-density lipoprotein cholesterol (HDL–C) (121). It has recently been shown that the loss of muscle mass is concomitant with fat mass gain (122). On the other hand, the type of dyslipidemia associated with sarcopenia in the elderly has not been yet adequately investigated or clearly defined (120). Serum TG and HDL-C concentrations have been shown to be independently associated with sarcopenic obesity (120). The evaluation of the lipid profile in a group of 84 elderly patients by measurement of multiple indices (total cholesterol TC, HDL-C and its intermediate HDL2, HDL3, low-density lipoprotein cholesterol (LDL-C), very low-density lipoprotein (VLDL), intermediate-density lipoprotein (IDL), LDL-particles (LDL-P), lipoprotein(a) and remnant-like particle cholesterol (RLP-C) showed that the presence of sarcopenia was associated mainly with changes in VLDL and RLP-C. The authors suggest that increased skeletal muscle mass leads to improvements in energy metabolism by promoting triglyceride-rich lipoprotein hydrolysis through an increase in the circulating levels of lipoprotein lipase (LPL) and its correlated enzyme for lipolysis glycosylphosphatidylinositol anchored high-density lipoprotein binding protein 1 (GPIHBP1) and vice versa in conditions of decreased muscle mass (120).

A recent systematic review demonstrates a correlation between low HDL-C levels and disease severity in SARS-CoV-2 infection. Potential mechanisms for the low HDL-C levels and reduced functionality in SARS-CoV-2 patients are not clearly defined. The “cytokine storm” seems to have a definite role in the immune-mediated inflammatory dyslipoproteinemia, that leads to lower HDL-C levels (123). Such HDL concentration reduction may have an impact not only on the reverse cholesterol transport but also on other relevant functions such as decreasing inflammation in immune effector cells and reducing endothelial responses (124).

To the best of our knowledge, only one experimental BR evaluated the impact of inactivity, during hypoxia, on the circulating levels of enzymes involved in lipid metabolism. Conditions of low oxygen pressure have been shown t*o cause a* significant increase in circulating Hepatic lipase (HL) and decrease in Lipoprotein Lipase (LPL) levels HL changes HDL2 to HDL3, The latter is quickly removed from the circulation by the liver (28). LPL is involved in the hydrolysis of the triglycerides transported in chylomicrons and VLDL, a lower concentration of the enzyme may reduce triglyceride clearance and increase their plasma levels. Hypoxia, associated with lower total HDL-C and HDL2-C sub-fractions, may therefore favor atherogenesis and increase CV risk.

All these findings suggest that in COVID-19 patients HDL protein composition and function are disrupted. This implies changes not only in the quantity but also in the quality of HDL-C. Moreover, these data reveal that low HDL-C levels could be added to the list of the well-known risk factors for COVID-19 severity (125). Moreover, given the well-known ability of these lipoproteins to normalize dyslipidemia and attenuate the inflammatory cascade, experimental and clinical studies have been conducted that have shown that HDL infusion may ameliorate endothelial function and reduce oxidative stress, inflammation and platelet aggregation (124). Nevertheless, further studies are required to better understand the role of HDL dysfunction in COVID-19 patients in order to provide tailored therapies.

### Muscle and Poly-Unsaturated Fatty Acids (PUFAs)

Omega-6 (n-6) and Omega-3 (n-3) PUFA series are known to be involved, respectively, in up-regulation and down-regulation of the inflammatory response (126).

Experimental studies have shown that both short and long-term bed rest are associated with increased membrane content of the pro-inflammatory n-6 arachidonic acid (28, 41) and a corresponding decrease in anti-inflammatory omega 3 fatty acids (alfa-linolenic and eicosapentaenoic acid, EPA). These data have been associated with a reduction in Δ5 desaturase enzymatic activity, influenced also by insulin (41). These results have been confirmed also in combined hypoxia and bed rest studies (28).

To date only one double blind, randomized clinical trial has been conducted to evaluate the effect n3-PUFA supplementation in critically ill SARS-CoV-2 patients. The Authors found that omega-3 administration improved the levels of several parameters of respiratory and renal function (123). Besides their anti-inflammatory effects, n3 PUFAs supplementation improved the anabolic efficiency of protein nutrition and exercise in elderly individuals and in patients with chronic diseases or cancer (23). Nevertheless, no other studies have yet confirmed these results.

Fatty acid (FA) composition in cell membrane phospholipids is strongly associated with systemic inflammatory response and insulin resistance (127–129). Bed rest and hypoxia additively decrease the delta-5 desaturase index, a key enzyme in the PUFA cascade and an established biomarker of insulin resistance (28, 41). Such a reduction, lowering PUFA within cell membranes, can modify insulin sensitivity (41).

It has been recently demonstrated that development of insulin resistance during SARS-CoV-2 infection is probably the mechanism leading to hyperglycemia development. This occurs through infectious insult to adipose tissue with aberrant secretion of adipokines by adipocytes. These data could have significant relevance not only for the management of hyperglycemia during infection, but also for the general understanding of the pathogenesis of severe COVID-19 (130). Moreover, insulin resistance may further decrease HDL plasma levels (131) worsening the previously described glucose and lipid homeostasis impairment.

### Sarcopenia in the Long COVID Syndrome

The long-term consequences of the SARS-CoV-2 infection remain largely unclear. Indeed, knowledge acquired in these 2 years of pandemia allows to differentiate acute COVID-19 from the SARS-CoV-2 infection sequelae and the Long COVID Syndrome (132). Patients with acute COVID-19 manifest signs and symptoms up to 4 weeks after the onset of the infection; Patients with COVID-19 sequelae are often elderly adult males with associated comorbidities who developed a severe acute SARS-CoV-2 infection leading to structural systemic damage (132). Patients with Long COVID may develop multi-organ symptoms that persist 4–12 weeks after the acute phase of illness, such as fatigue, post-exertional malaise, cognitive dysfunction, and limitation of functional capacity. According to the data available so far, at least 10% of acute COVID-19 survivors develop Long COVID (132).

COVID-19 ICU patients show significant reductions in skeletal muscle mass and strength during their hospital stay (133, 134). Indeed, after 10 days patients showed a 30% loss in rectus femoris cross-sectional area, a lower thickness (of almost 20%) of the quadriceps muscle (133) and weakness during knee-extensor and arm-flexor tests (135). The impairment in quadriceps strength and in general of muscle strength was significantly associated with a longer hospital stay (136). Low muscle mass is an independent risk factor for mortality (136). Not surprisingly, SARS-CoV-2 infected sarcopenic patients need a twice-long hospital stay and have an eight times higher mortality rate than non-sarcopenic subjects (137).

SARS-CoV-2 infection induces a status of muscle weakness similar to that seen in ICU-acquired weakness, which results in a long-term physical disability. Although comparative studies are required, the skeletal muscle alterations in hospitalized COVID-19 patients can be included in the broad category of ICU-acquired weakness.

Hospitalized patients (particularly ICU patients) require a long-term rehabilitation after discharge (138). There is a need for long-term follow-up studies on persistent symptoms, lung function, physical, and psychological derangement in discharged, patients. Some of the myokines, synthetized after physical exercise can reduce systemic inflammation through their cross talk between muscle and other organs (133).

Optimal management strategies to improve fatigue and exercise capacity in both acute- and long-COVID-19 patients are still debated (132). Recommendations for physical therapy management of COVID-19 patients have been published (139) but are still suboptimal (133, 140). Promoting immediate ambulation of ICU patients has proven to be feasible and has positive effects (133). For patients in whom this is not possible, the potential efficacy of neuromuscular electrical stimulation has been shown (141). Table 1 report a pragmatic review of potential treatments for sarcopenia and weakness in patients with post-acute sequelae of COVID-19 (142–145). Rehabilitation, i.e., regular exercise, has been shown to be effective in preventing and ameliorating the long-term debilitating effects of critical illness myopathy (146). Furthermore, physiotherapy after acute COVID-19 infection can also support the respiratory and physical rehabilitation of patients with COVID-19 (147, 148).

**Table 1:** Potential treatments of sarcopenia and weakness in patients with post-acute sequelae of COVID-19.

**Reference**	**Experimental design**	**Patients**	**Covid status**	**Interventions**	**Results**
Nambi et al. (142)	RCT	Community-dwelling sarcopenic old men	Post COVID-19	Low/high-intensity aerobic exercise (8 weeks).	Improves muscle strength, kinesiophobia and quality of life
Rodriguez-Blanco et al. (143)	RCT	Home confined patients	Moderate or acute COVID-19	Tele-rehabilitation program (one-week)	Valuable, secure, and achievable during disease.
Mohamed et al. (144)	RCT	Hospitalized Patients	Mild or moderate COVID-19	Moderate aerobic exercise (2 weeks)	Reduces severity and progression of COVID-19 associated disorders and improves quality of life.
Tang et al. (145)	Observational longitudinal study	Discharged patients	Post COVID-19	Liuzijue exercise (4 weeks)	Enhanced quality of life and functional capacity

*RCT, Randomized Controlled Trial*.

## Conclusions

**Figure 1:**
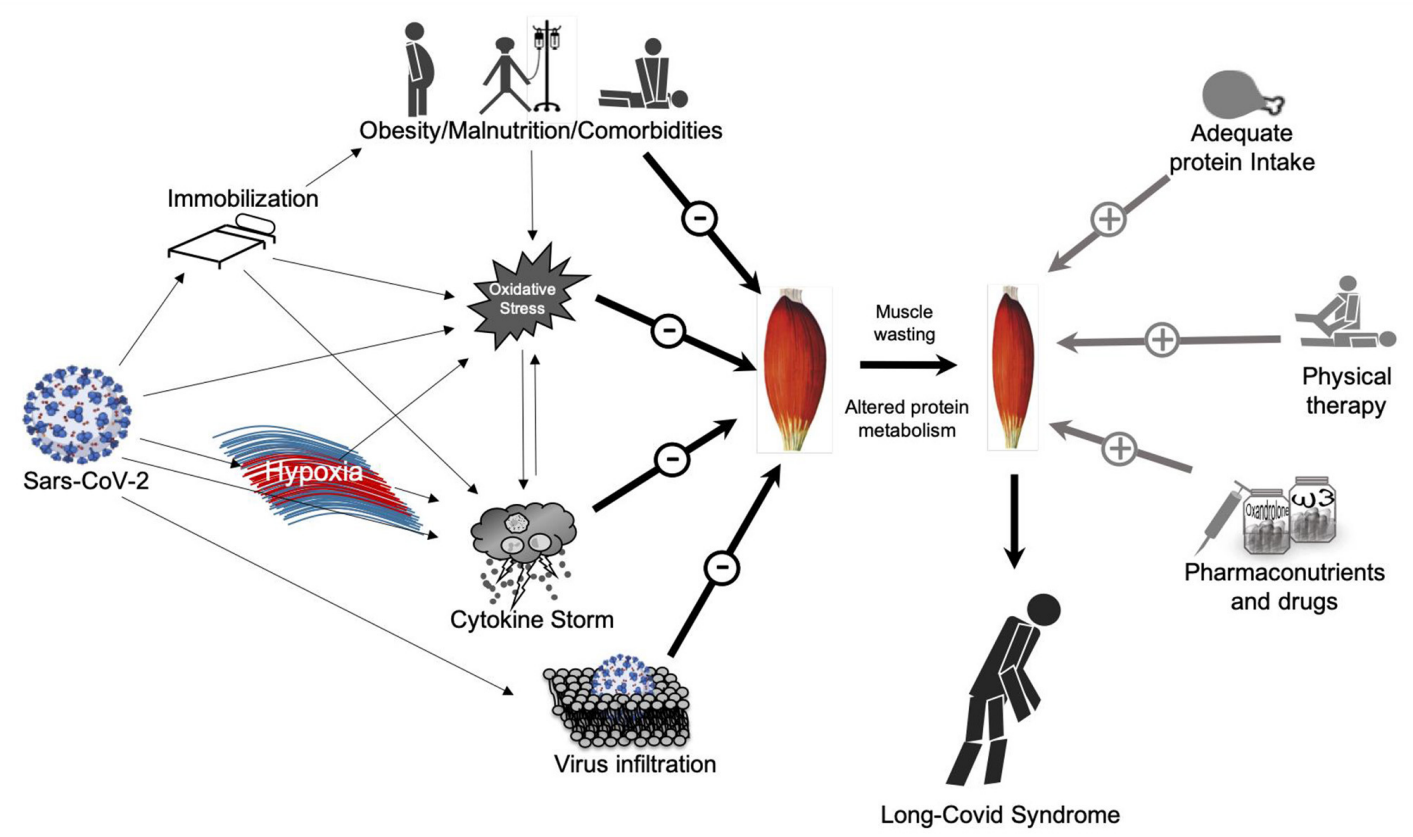
Direct and indirect effects of Sars-CoV-2 on skeletal muscle mass and possible countermeasures. On the left side: Direct and indirect effects of Sars-CoV-2 on muscle mass potentially lead to muscle wasting and the so called “long-Covid Syndrome”; on the right-side: possible countermeasures to avoid or at least maintain muscle mass quality and quantity.

SARS-CoV-2 infection is a multifactorial disease in which a number of detrimental factors interplay together in a way that can lead to acute severe COVID-19, ICU hospitalization and possibly long-COVID-19 syndrome after discharge (Figure 1).

Reduced muscle strength is a prominent symptom of acute COVID-19 and long-COVID syndrome, and malnutrition, limited physical activity and some drugs may further reduce muscular function and add to the pathogenicity of the virus.

We tried to shed light on both direct and indirect potential detrimental consequences of SARS-CoV-2 on skeletal muscle mass, focusing on the effect of hypoxia. As for other viral pulmonary diseases, hypoxia seems to play an important role in COVID-19 exacerbation, leading to a more severe prognosis. Moreover, hypoxia is an important driver of symptoms in SARS-CoV-2 infection particularly for those related to muscle wasting. Hypoxia, especially when associated with bed rest, seems to activate a complex crosstalk of key signaling pathways of oxidative stress and inflammation leading to subsequent lipid and glucose metabolism derangements.

One limitation of this manuscript is the lack of data about the direct viral infiltration of SARS-CoV-2 in skeletal muscle. Indeed, future studies across larger cohorts are required to completely understand the molecular basis of COVID-19-related skeletal muscle alterations for a more precise and customized interventions aimed at improving the patient's clinical symptoms and prognosis.

## Author Contributions

GB and FD: conceptualization and supervision. PS, GB, and FD: validation. FD, US, NF, AN, FM, and PV: writing—original draft preparation. NF, FD, and GB: writing—review and editing. All authors have read and agreed to the published version of the manuscript.

## Conflict of Interest

The authors declare that the research was conducted in the absence of any commercial or financial relationships that could be construed as a potential conflict of interest.

## Publisher's Note

All claims expressed in this article are solely those of the authors and do not necessarily represent those of their affiliated organizations, or those of the publisher, the editors and the reviewers. Any product that may be evaluated in this article, or claim that may be made by its manufacturer, is not guaranteed or endorsed by the publisher.
